# Missed case of sinus venosus atrial septal defect post coronary artery bypass grafting

**DOI:** 10.1186/1749-8090-9-91

**Published:** 2014-05-19

**Authors:** Sudeep Das De, Winn Maung Maung Aye, Sriram Shankar

**Affiliations:** 1 National University Health System, 1E Kent Ridge Road, Kent Ridge 119228, Singapore

**Keywords:** Sinus venosus atrial septal defect, Partial anomalous pulmonary venous drainage, CABG

## Abstract

We report a case of a 41-year-old Indian man who initially underwent an emergency coronary artery bypass grafting surgery (CABG) after presenting with an anterolateral myocardial infarction.

Post-operatively he developed progressively worsening symptoms of right heart failure with increasing abdominal distension and lower limb swelling. Clinically, the patient was in NYHA class 4 heart failure.

He was admitted multiple times for the treatment of his heart failure, which was recalcitrant to diuretic therapy.

He subsequently underwent an MRI scan, which revealed near transmural myocardial infarction involving mainly the left side of the heart. The right atrium and ventricle were grossly dilated, with moderate to severe right ventricular systolic dysfunction. A sinus venosus atrial septal defect with right-sided partial anomalous pulmonary venous drainange (PAPVD) was noted. He subsequently underwent surgery to repair the sinus venosus atrial septal defect (ASD) as well as re-route the PAPVD to the left atrium (LA). He was discharged on post-operative day 19 with oral diuretics.

On follow-up at 1 month, the patient's symptoms had resolved and his clinical status corresponded to NYHA class 1–2.

## Background

Atrial septal defect (ASD), one of the commonest congenital heart lesions, is asymptomatic in most cases and therefore remains undiagnosed until adulthood in a significant number of patients.

Adults with congenital heart defects represent a growing population. Atherosclerotic coronary artery disease may be seen during repair of adult congenital heart disease (ACHD), with the most common cardiac diagnosis being secundum atrial septal defects [1]. Here we report a case of a patient diagnosed incidentally with a sinus venosus ASD, after he underwent a CABG for a myocardial infarction. This was missed at the initial operation and only diagnosed post-operatively when the patient developed symptoms of right heart failure.

### Case presentation

A 41-year-old Indian man presented with sudden onscet of chest pain in February 2011 and was diagnosed with an anterolateral myocardial infarction (MI); prior to the event, he was completely asymptomatic. The coronary angiogram revealed an occluded ostial and proximal left anterior descending (LAD) artery. After a failed percutaneous coronary intervention (PCI), he underwent emergency coronary artery bypass grafting (CABG) involving the left internal mammary artery (LIMA) to the LAD.

Post-operatively he developed progressively worsening symptoms of right heart failure with increasing abdominal distension and lower limb swelling. Clinically, the patient was in NYHA class 4 heart failure. The 2D echocardiogram revealed an ejection fraction of 10% and a pulmonary artery systolic pressure (PASP) of 40 mmHg.

He was admitted multiple times for the treatment of his heart failure, which was recalcitrant to diuretic therapy. He was eventually offered a heart-lung transplant to alleviate his symptoms.

In October 2011, he was admitted to another institution for further re-evaluation of his symptoms.

A repeat 2D echocardiogram revealed severe biventricular dysfunction with a left ventricular ejection fraction (LVEF) of 12%, a PASP of 38 mmHg as well as severe tricuspid regurgitation (TR). There was a thinned out ventricular septum with a possible left ventricular (LV) apical aneurysm.

Multiple attempts to further evaluate the apical aneurysm or additional intracardiac lesions with a cardiac MRI failed, as the patient was unable to lie supine because of his severe dyspnoea.

A decision was made to insert a peritoneal dialysis catheter to alleviate his abdominal distension.

**Figure 1:**
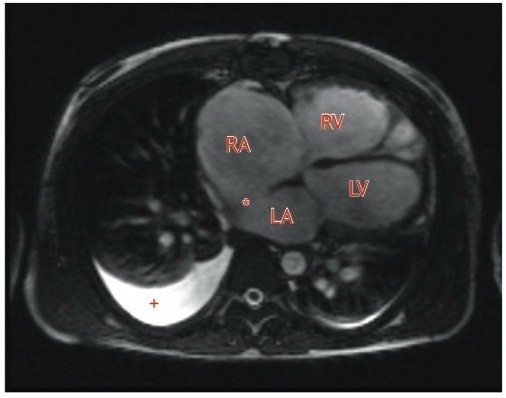
Axial T2-weighted MRI showing the grossly dilated right heart and the sinus venosus atrial septal defect (*). RA- right atrium, RV-right ventricle, LA-left atrium, LV–left ventricle. Right sided pleural effusion (+).

He subsequently tolerated an MRI scan, which revealed near transmural myocardial infarction involving mainly the LAD and left circumflex (LCx) territories; the segments supplied by the right coronary artery (RCA) were viable. The right atrium and ventricle were grossly dilated, with moderate to severe right ventricular systolic dysfunction. A sinus venosus atrial septal defect with right-sided partial anomalous pulmonary venous drainage (PAPVD) was noted. There was left to right shunting (QPQS 4.04) with moderate to severe tricuspid regurgitation; no LV apical aneurysm was detected (Figure 1).

A redo-median sternotomy was performed, followed by clearing of dense adhesions. Cardiopulmonary bypass (CPB) was commenced with bicaval venous cannulation (high SVC cannula) and aortic cannulation in the ascending aorta. A LV vent was inserted through the LA. The SVC and IVC were snared followed by opening of the right atrium (RA).

Intra-operative findings were that of a high sinus venosus ASD with PAPVD (RSPV X 2). The sinus venosus ASD was repaired with an autologous pericardial patch and the PAPVD was re-routed to the LA with continuous non-absorbable sutures. As the PA pressure was low, a De Vega annuloplasty of the triuspid valve was performed with non-absorbable pledgeted sutures.

The heart was oedematous after weaning from CPB. The sternotomy wound was left open and was closed on the second post-operative day.

A post-operative echo revealed a visual EF of 30%, a PASP of 59 and moderate tricuspid regurgitation with no residual shunt.

He developed a right-sided pleural effusion on post-op day 6 for which a pigtail catheter was inserted. The pleural effusion resolve and the post-operative period was otherwise unremarkable.

He was discharged on post-operative day 19 with oral diuretics.

On follow-up at 1 month post-operatively, the patient's symptoms had resolved and his clinical status corresponded to NYHA class 1–2.

## Conclusions

There have been reports of ASDs being diagnosed incidentally on intra-operative TEE during coronary artery surgery [2,3]. These have been repaired concomitantly with coronary artery bypass grafting (CABG) with successful outcomes.

Here we report a case of a sinus venosus ASD diagnosed after the initial CABG. The patient had no symptoms of his ASD prior to the myocardial infarction. This highlights the fact that ASDs may be completely asymptomatic up to an acute coronary event. In this case his anterolateral MI exacerbated the left to right shunting through the ASD causing right ventricular overload and subsequently right sided heart failure. The likely reason that the LVEF improved after reducing the left to right shunt can be attributed to the fact that more blood would flow from the left atrium into the left ventricle and thus increase the LV preload and LVEF.

We must have a high index of suspicion to the presence of concomitant congenital defects and coronary artery disease especially when clinical and echo findings are not congruent with the initial diagnosis. In this case the MI involved the left side of the heart but the symptoms were predominantly those of right-sided heart failure. Intra-operatively, any unexplained dilatation of the right heart should prompt the question of a possible left to right shunt at the atrial level including a sinus venosus defect, although this was not noted during the initial surgery in this case.

There is an increasing role for the use of MRIs as a gold standard in evaluating cardiac function as well as anatomy. We also recommend the routine use of intra-operative TEE during coronary artery bypass grafting as this has a higher sensitivity in detecting high sinus venosus ASDs compared to standard 2D Echos [4,5].

### Consent

Written informed consent was obtained from the patient for publication of this Case report and any accompanying images. Written informed consent was obtained from the patient for publication of this case report and any accompanying images. A copy of the written consent is available for review by the Editor-in-Chief of this journal.

## Abbreviations

ASD: Atrial septal defetct; PAPVD: Partial anomalous pulmonary venous drainage; MI: Myocardial infarction; LA: Left atrium; RA: Right atrium; LV: Left ventricle; RV: Right ventricle; SVC: superior vena cava; IVC: Inferior vena cava; CABG: Coronary atery bypass grafting; PASP: Pulmonary artery systolic pressure; NYHA: New York Heart association.

## Competing interests

The authors declare that there are no financial or non-financial competing interests.

## Authors’ contributions

SS was the primary surgeon for the patient, WM was the first assistant and SDD was the second assistant. All the authors were involved in the post-operative care of this patient. SDD wrote the article and edited the image. All authors read and approved the final manuscript.

## References

[B1] Webb G Gatzoulis MA Atrial septal defects in the adult: recent progress and overview Circulation 2006 October 10 9 15 1645 53 10.1161/CIRCULATIONAHA.105.592055 17030704

[B2] Shimoyama Y Sawai T Nakahira J Minami T [Atrial septal defect of sinus venous type diagnosed by transesophageal echocardiography intraoperatively] Masui 2009 9 2 189 192 19227174

[B3] Fores B Olmos M de SA O Gonzalez JA [Incidental diagnosis of an ostium-secundum-type interatrial communication during coronary surgery] Rev Esp Anestesiol Reanim 2003 9 6 299 302 12940220

[B4] Taylor AM Stables RH Poole-Wilson PA Pennell DJ Definitive clinical assessment of atrial septal defect by magnetic resonance imaging J Cardiovasc Magn Reson 1999 9 1 43 7 10.3109/10976649909080832 11550340

[B5] Prompona M Muehling O Naebauer M Schoenberg SO Reiser M Huber A MRI for detection of anomalous pulmonary venous drainage in patients with sinus venosus atrial septal defects Int J Cardiovasc Imaging 2011 March 9 3 403 12 10.1007/s10554-010-9675-3 20686854

